# Characterization and comparative analysis of the complete mitochondrial genome of *Ferula sinkiangensis* (K. M. Shen, 1975) (Apiales: Apiaceae)

**DOI:** 10.1080/23802359.2026.2621449

**Published:** 2026-04-20

**Authors:** Xinqiao Deng, Congzhao Fan, Wendan Song, Zhancang Ma, Guoping Wang, Ping Yan

**Affiliations:** a Key Laboratory of Xinjiang Phytomedicine resource and Utilization, Ministry of Education, College of Life Sciences, Shihezi University, Shihezi, Xinjiang, China; b Xinjiang Key Laboratory of Chinese Materia Medica and Ethnic Materia Medica, Xinjiang Institute of Materia Medica, Urumqi, Xinjiang, China

**Keywords:** *Ferula sinkiangensis*, mitochondrial genome, apiaceae, phylogenetic analysis

## Abstract

This study presents the first complete mitochondrial genome of *Ferula sinkiangensis*, a medicinal plant formally recognized in the Chinese Pharmacopoeia since 1977. The assembled mitogenome is 416,958 bp in length, encoding 97 genes, and exhibits a structurally complex architecture characterized by 28 large repeat pairs. Phylogenetic analysis robustly places *F. sinkiangensis* within a fully supported clade. These findings underscore the necessity of long-read sequencing to fully resolve its genomic isoforms.

## 1. Introduction

*Ferula* L. is a major genus in the Apiaceae family, comprising approximately 223 species primarily distributed across Central Asia, the Mediterranean region, and Southern Europe (Pimenov and Leonov 1993; Plants of the World Online 2025; Yang et al. 2025). *Ferula* species hold significant medicinal value and have been traditionally used to alleviate stagnation, reduce inflammatory swelling, and act as anthelmintics (Chinese Pharmacopoeia Commission 2025). Certain species produce aromatic resins that are processed into the traditional medicine known as ‘a wei’. Historically, China imported *Ferula* species from West Asia; however, native species have also been identified in Xinjiang. This led to the inclusion of *F. sinkiangensis* K. M. Shen in the Chinese Pharmacopeia since 1977 (Wang Wenjie et al. 2005).

*Ferula sinkiangensis* (Plants of the World Online, n.d.), a perennial herb endemic to Xinjiang, flowers from April to May and fruits from June to July. It typically grows on clay-loam slopes at elevations of 700–1,000 m (Commissione Redactorum Florae Xinjiangensis 2011). Its monocarpic growth habit, in which the plant dies after fruiting, combined with overharvesting, has rendered wild populations critically endangered, as indicated by its designation as a National Grade II Protected species and an IUCN Critically Endangered species (National Forestry and Grassland Administration, 2021).

Although the mitochondrial genome of *F. sinkiangensis* (GenBank accession OK585063, released on 13 November 2021) has been previously published and used in broader analyses (Jo et al. 2024), a detailed characterization of its architectural features, including repetitive elements and recombination potential, along with a robust phylogenetic analysis using multiple inference methods, is lacking. This study provides a comprehensive analysis and offers new insights into the genomic organization and evolutionary relationships of this endangered species.

## 2. Materials and methods

### 2.1. Plant collection

**Figure 1.**
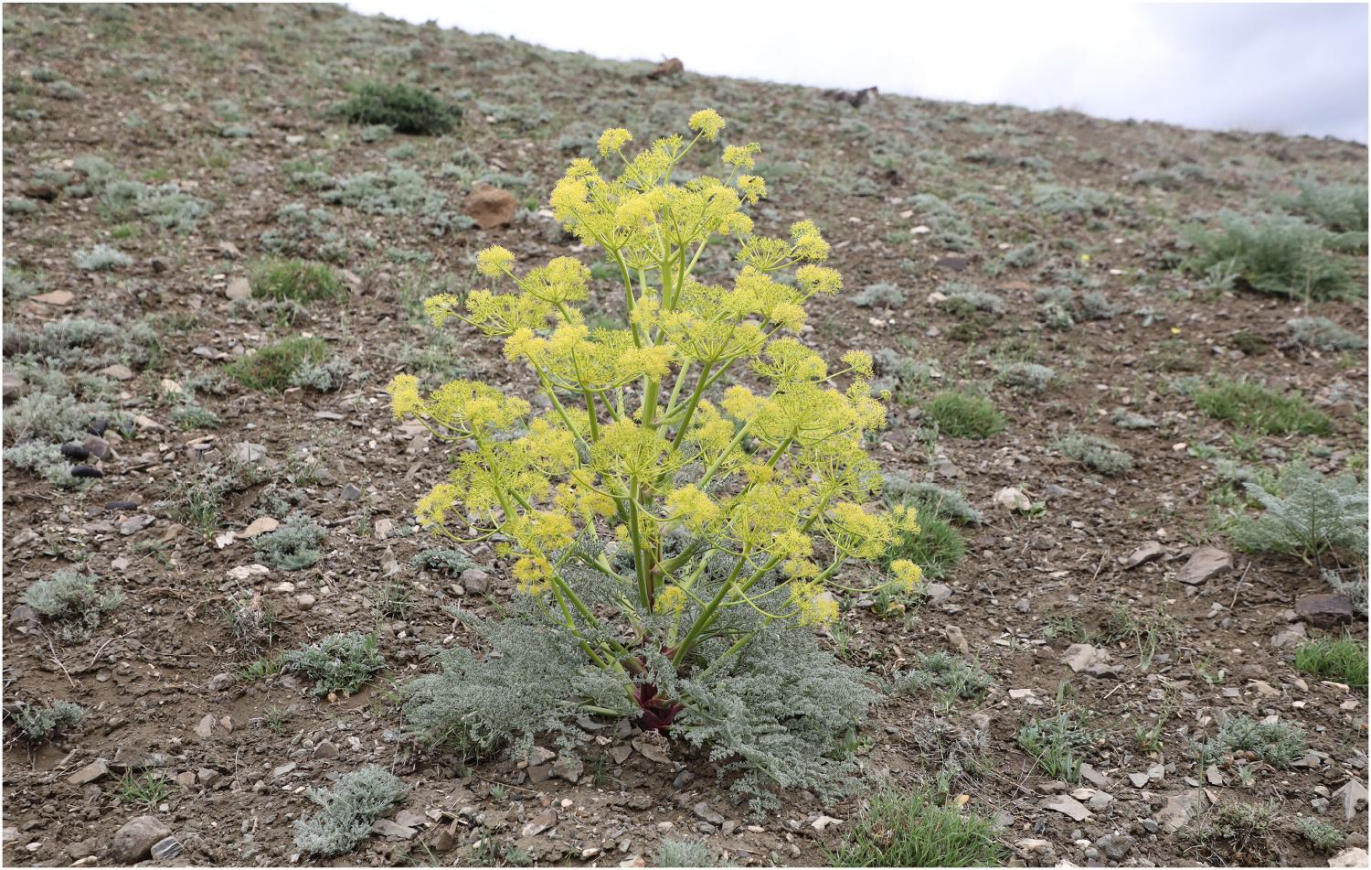
Species reference image of *F. sinkiangensis*. The photograph was taken by the author (Congzhao Fan).

A cultivated specimen of *F. sinkiangensis* was collected from the wild-tending base of *F. sinkiangensis* in Yining County (82.0574° E, 43.6743° N), Xinjiang, China (Figure 1). This site is managed by the Xinjiang Institute of Materia Medica for conservation and research purposes. Specific geographic coordinates are provided because they correspond solely to this cultivated accession and do not threaten the protection of wild populations. The specimen was deposited at the Xinjiang Institute of Materia Medica Herbarium (XTNM; Congzhao Fan; fcz_840701@163.com) under the accession number 654021240525001LY. This study adhered to all relevant institutional, national, and international guidelines.

### 2.2. DNA extraction, sequencing, assembly, and annotation

Total DNA was extracted from fresh leaves using a DNeasy Plant Mini Kit (Qiagen, Valencia, USA). Sequencing was performed using the Illumina HiSeq X Ten platform (Illumina, San Diego, CA, USA). Sequence data corresponding to the chloroplast genome have been previously published. Because the raw sequencing data are identical, the current study used the same BioProject, SRA, and Bio-Sample accession numbers as the previous publication (Fan et al. 2021; https://www.ncbi.nlm.nih.gov/pmc/articles/PMC8143607). Raw data were assessed for quality assessment using FastQC v0.11.8 (http://www.bioinformatics.babraham.ac.uk/projects/fastqc/) and MultiQC v1.31 (http://multiqc.info) (Supplementary Figure 1-3), followed by adapter trimming and quality filtering using Fastp v0.19.5 (Chen et al. 2018). Potential plastid-derived sequences were removed using BWA v0.7.17 (Li and Durbin 2009). De novo assembly of the mitochondrial genome was conducted using GetOrganelle v1.6.4 (Jin et al. 2020) with a k-mer size of 117 and an insert size of 350 bp, guided by seed sequences from the *cox1, cox3*, and *matR* genes of *Daucus carota* subsp. *sativus* (GenBank accession JQ248574) (Iorizzo et al. 2012) The sequencing depth and coverage statistics are illustrated in Supplementary Figure 4. Genome annotation was performed using PMGA (http://47.96.249.172:16084/index.html) (Li et al. 2025) and subsequently refined using PMGmap (http://47.96.249.172:16086/home/) (Zhang et al. 2024) for structural visualization and identification of cis- and trans-splicing genes. Long repeats were identified using REPuter (Kurtz et al. 2001), and large repetitive sequences (≥1000 bp) were detected using BLAST+ v2.9.0 (Camacho et al. 2009). The resulting coverage distribution is shown in Supplementary Figure 5.

**Figure 2.**
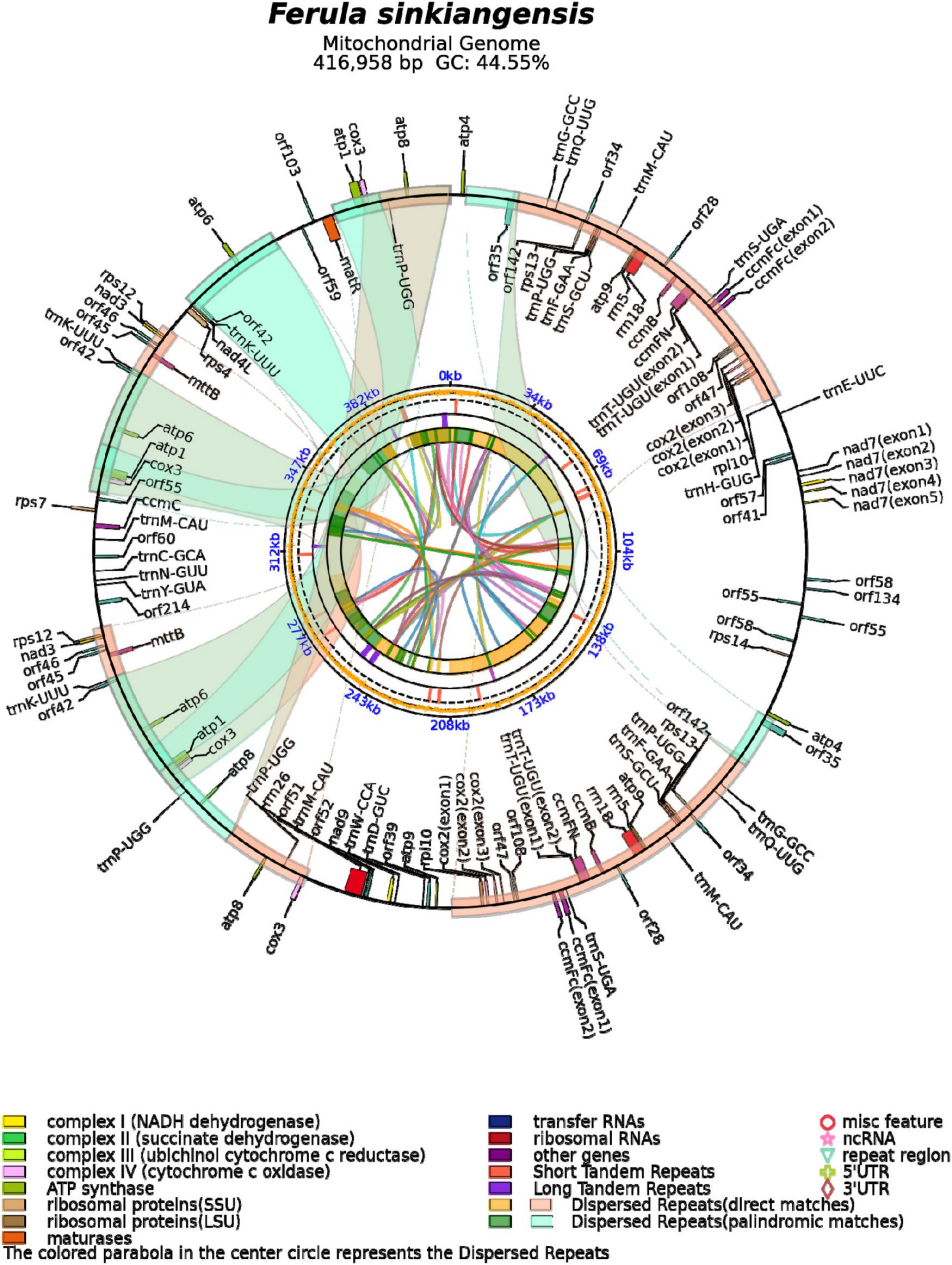
Circular map of *F. sinkiangensis* mitogenome. Genes transcribed in the clockwise direction are shown on the outer circle, whereas those transcribed in the counterclockwise direction are shown on the inner circle. Genes are color-coded according to their functional category.

**Figure 3.**
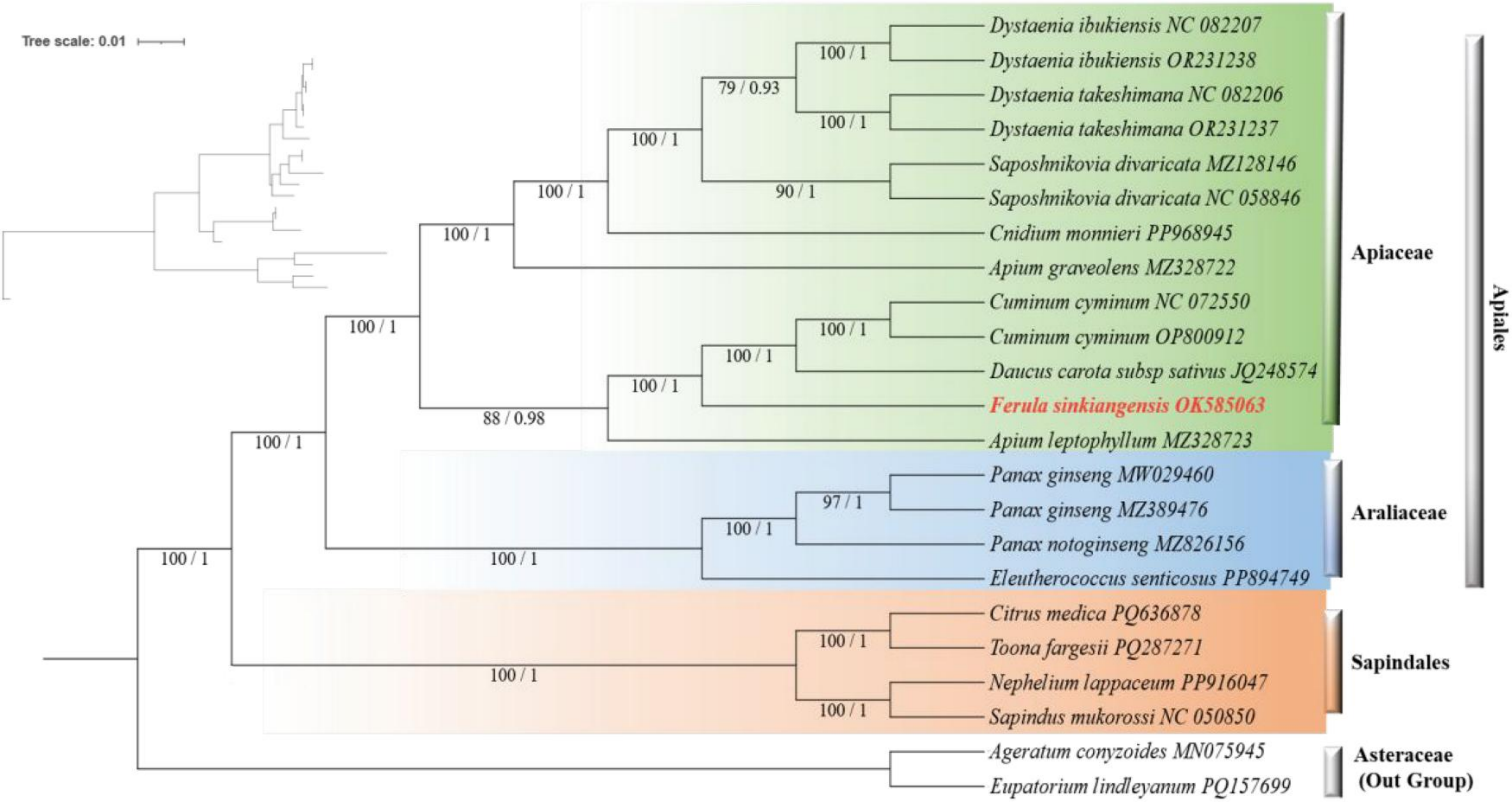
Phylogenetic relationships of *F. sinkiangensis* and related taxa inferred from complete mitochondrial genome sequences using maximum likelihood (ML) and bayesian inference (BI). The figure comprises two panels: a branch length scale and the detailed phylogenetic tree. Branch support values are indicated as ML bootstrap support/BI posterior probability (ML-BS/BI-PP). Outgroups are represented by species from sapindales and asteraceae. The GenBank accession numbers and sources for all sequences are as follows: *Dystaenia ibukiensis* (NC_082207), *dystaenia takesimana* (NC_082206), *dystaenia ibukiensis* (OR231238), *dystaenia takesimana* (OR231237), *cnidium monnieri* (PP968945), *cuminum cyminum* (NC_072550), *cuminum cyminum* (OP800912), *saposhnikovia divaricata* (NC_058846), *saposhnikovia divaricata* (MZ128146), *apium leptophyllum* (MZ328723), *apium graveolens* (MZ328722), *daucus carota* subsp. *sativus* (JQ248574) (Iorizzo et al. 2012), *panax ginseng* (MW029460) (Woojong Jang et al. 2020), *panax ginseng* (MZ389476), *panax notoginseng* (MZ826156), *eleutherococcus senticosus* (PP894749), *citrus medica* (PQ636878), *sapindus mukorossi* (NC_050850), *toona fargesii* (PQ287271), *nephelium lappaceum* (PP916047), *eupatorium lindleyanum* (PQ157699), *ageratum conyzoides* (MN075945) (Luo and Pan 2019). The target species, *F. sinkiangensis* is highlighted in bold red.

### 2.3. Phylogenetic analysis

To investigate the phylogenetic relationship of *F. sinkiangensis*, 23 mitochondrial genomes were retrieved from the NCBI database (Supplementary Table 1). The 14 shared protein-coding genes (Supplementary Table 2) were identified using PhyloSuite v1.2.3 (Xiang et al. 2023), aligned using MAFFT v7.526 (Katoh and Standley 2013), and subsequently concatenated using PhyloSuite v1.2.3. Using *Eupatorium lindleyanum* (PQ157699) and *Ageratum conyzoides* (MN075945) (Luo and Pan 2019) as outgroups, phylogenetic analyses were conducted with the following methods (Kubatko and Degnan 2007): (1) Maximum Likelihood (ML) analysis performed in IQ-TREE v2.2.0 (Minh et al. 2020) under the GTR+F + I + G4 model selected by ModelFinder and 1000 bootstrap replicates (Kalyaanamoorthy et al. 2017); (2) A partitioned ML analysis based on gene and codon partitions defined by PartitionFinder2 (Lanfear et al. 2017) and executed in IQ-TREE v2.2.0; and (3) Bayesian Inference (BI) performed in BEAST2 v2.6.3 (Bouckaert et al. 2014) under a GTR + Γmodel with an uncorrelated lognormal relaxed clock and a Yule tree prior, run for 10 million MCMC generations until all ESS values exceeded 200. The resulting trees were visualized using iTOL (https://itol.embl.de/).

All software versions and primary command lines used in the analyses are listed in Supplementary Table 3.

## 3. Results

Approximately 65,371,722 raw reads were generated and processed using quality control filters (Supplementary Table 4, Supplementary Figure s 6-7). A representative structure of the *F. sinkiangensis* mitochondrial genome was assembled and annotated (Figure 2). The assembled sequence was 416,958 bp long, with a GC content of 44.55% and an asymmetric base composition (A: 27.64%, T: 27.81%, G: 22.05%, and C: 22.50%). Annotation revealed a total of 97 genes, comprising 62 protein-coding genes (31 unique), 31 transfer RNA genes (18 unique), and four ribosomal RNA genes. Of these, six cis-splicing genes and three trans-splicing genes were identified (Supplementary Figure 8 and 9). Consistent with the complexity of the plant mitochondrial genome (Kozik et al. 2019), short-read data alone are insufficient to fully resolve the complete spectrum of alternative isoforms. Therefore, the present assembly should be considered a representative structure rather than a single ‘master circle’ conformation. A total of 28 repeat pairs ≥1 kb were detected, including 14 repeats ≥10 kb (maximum length: 63,114 bp; Supplementary Table 5 and Supplementary Figure 10). To assess recombination potential, we mapped large repeats to construct a recombination model (Supplementary Figure 11). The validation of putative junctions by paired-end read bridging, combined with the clustering of repeat boundaries, revealed four primary hotspots (Supplementary Figure 12; Supplementary Table 6), indicating active regions of elevated recombination activity that shape the mitochondrial genome architecture. Genome-wide coverage profiles are shown in Supplementary Figure 5. Although short-read data robustly support recombination, long-read sequencing is required to fully resolve the alternative mitochondrial conformations.

Phylogenetic trees reconstructed using the ML (concatenated model), Partition Model (by gene and codon position), and BI methods yielded highly congruent topologies (Robinson-Foulds distance ≤ 2). Therefore, only the ML tree is presented (Figure 3), with node support values derived from ML bootstrap (ML-BS) and BI posterior probability (BI-PP) analyses. In this tree, *F. sinkiangensis* formed a strongly supported clade with *C. cyminum* and *D. carota* subsp. *sativus* (ML-BS = 100; BI-PP = 1.00). All nodes within this clade and the broader Apioideae lineage exhibited strong support (BS ≥ 94.2, PP = 1.00). The trees generated by the Partition Model and BI analyses are shown in Supplementary Figure 13 and 14, respectively.

## 4. Discussion and conclusion

This study offers a comprehensive characterization of the *F. sinkiangensis* mitochondrial genome, which has been publicly available in GenBank under accession number OK585063 since 2021. Assembly and annotation produced a representative model of the genome structure, revealing extensive repetitive sequences and recombination-active regions. These findings suggest a complex graph-like organization rather than a single predominant circular form.

Our assembly strategy and findings are consistent with the current methodological approaches and persistent challenges in plant mitogenomics, as outlined in a recent comprehensive review by Ni et al. (Ni et al. 2025). Ni et al. emphasized the prevalence of complex multichromosomal structures within plant mitochondria and the inherent limitations of short-read data in resolving them. Specifically, our identification of 28 large repeat pairs (≥1 kb), 14 of which exceed 10 kb, and the prediction of four primary recombination hotspots in *F. sinkiangensis* directly reflect the ‘graph-like’ genome architecture and repeat-driven structural dynamics as described by Ni et al. Although the Illumina- and GetOrganelle-based approaches yielded a robust assembly of a representative structure, they are inherently unable to resolve the alternative conformations generated by repeat-mediated recombination. This limitation directly corresponds to the emphasis of Ni et al. on the essential role of long-read sequencing in advancing from inferred recombination models to fully resolved haplotype sequences (Ni et al. 2025). Consequently, our study provides a concrete case that underscores the central argument presented by Ni et al. that for complex mitogenomes, short-read assemblies represent a necessary but intermediate step toward complete resolution.

In addition to its overall complexity, our annotation revealed specific structural features with potential evolutionary and functional significance. The mitogenome encodes 97 genes, including six cis-splicing and three trans-splicing genes that require particular attention. The presence of trans-splicing genes is characteristic of the highly fragmented mitochondrial genomes of some angiosperms and is often mechanistically associated with homologous recombination. In *F. sinkiangensis*, these trans-splicing events may be directly facilitated by the active recombination hotspots identified in this study, suggesting a tangible connection between the dynamic genome structure and the gene expression machinery. This abundance of repetitive DNA is not merely a structural curiosity; it serves as the primary driver of genomic plasticity and isoform diversity, thereby complicating genome assembly. Collectively, these features portray the *F. sinkiangensis* mitogenome as a highly plastic and recombinogenic system, whose structural biology is deeply intertwined with its repetitive landscape.

Phylogenetic analyses, using both partitioned ML and Bayesian methods, consistently supported a close relationship among *F. sinkiangensis*, *C. cyminum*, and *D. carota* subsp. *sativus*, thereby providing a robust evolutionary framework for this endangered medicinal plant.

Although assemblies generated from short-read sequencing offer valuable structural and comparative insights, they remain inherently limited in resolving the full spectrum of mitochondrial genomic isoforms. Future studies incorporating long-read sequencing will be essential for elucidating structural variations and haplotype diversity, thereby directly addressing the methodological challenges highlighted in a recent review.

Overall, the mitochondrial genome presented here offers a strong foundation for evolutionary studies, genetic conservation efforts, and taxonomic clarification within *Ferula* and the related taxa.

## Supplementary Material

Supplementary Materials Unmarked Version 1208.doc

## Data Availability

We confirm that the complete mitogenome sequence could be accessed using the NCBI GenBank accession number. OK585063 at https://www.ncbi.nlm.nih.gov. The associated BioProject, SRA, and BioSample numbers are PRJNA715948, SRR14018637, and SAMN18388803, respectively. Because the sample of *F. sinkiangensis* is the same, and we used a set of original data to analyze the chloroplasts and mitochondria genomes, this manuscript has the same BioProject, BioSample numbers, and Geo-coordinates as in the published article https://www.ncbi.nlm.nih.gov/labs/pmc/articles/PMC8143607/.
