# Supplementary material for: Characterization and comparative analysis of the complete mitochondrial genome of Ferula sinkiangensis (K. M. Shen, 1975) (Apiales: Apiaceae)
Source: Mitochondrial DNA B Resour. 2026 Apr 20;11(5):664–9. doi: 10.1080/23802359.2026.2621449 (PMC13097163; doi:10.1080/23802359.2026.2621449)
Supplement: Supplementary Materials Unmarked Version 1208.doc [file TMDN_A_2621449_SM2913.doc]

**Supplementary Materials**

**(Characterization and comparative analysis of the complete mitochondrial genome of *Ferula sinkiangensis* (K. M. Shen, 1975) (Apiales: Apiaceae))**

**Xinqiao Deng, Congzhao Fan, Wendan Song, Zhancang Ma, Guoping Wang, Ping Yan**

**
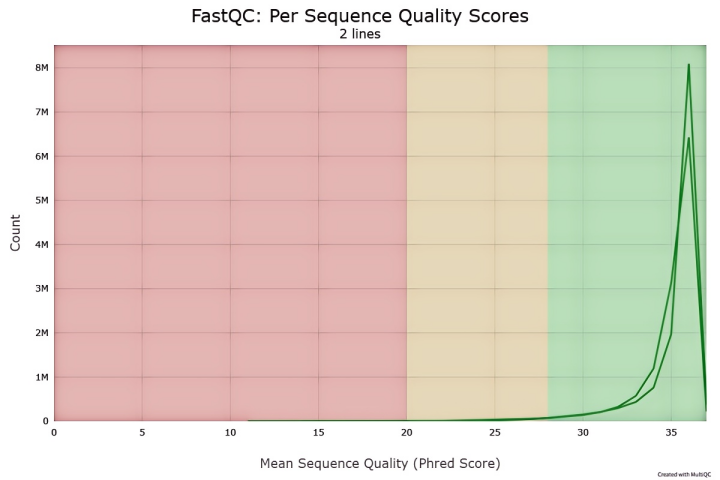
**

**Supplementary Figure 1.** Distribution of mean sequencing quality scores across base positions. The average quality scores for all samples remained consistently above the Q30 threshold (dashed line) throughout the entire read length (positions 1–150 bp), including the read-en


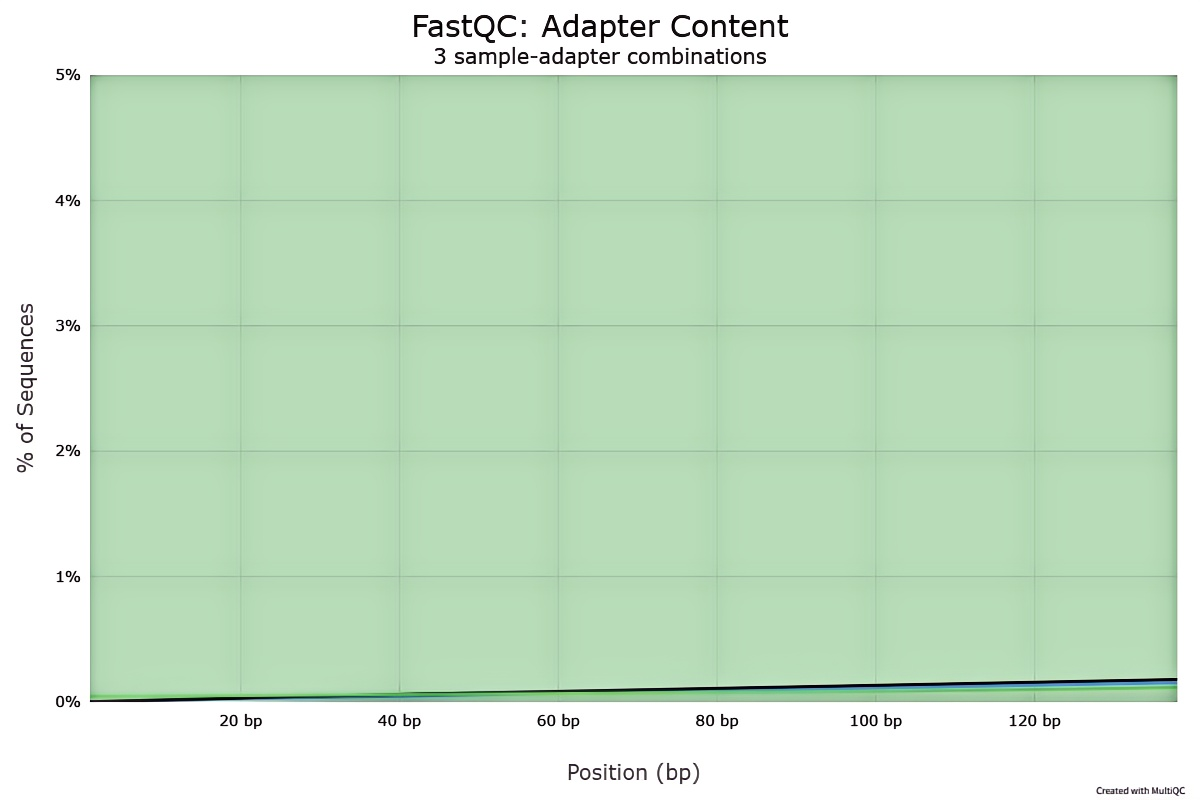


**Supplementary Figure 2.** Adapter content across read length. All samples exhibited minimal adapter contamination (<0.2%) throughout the sequenced fragment.


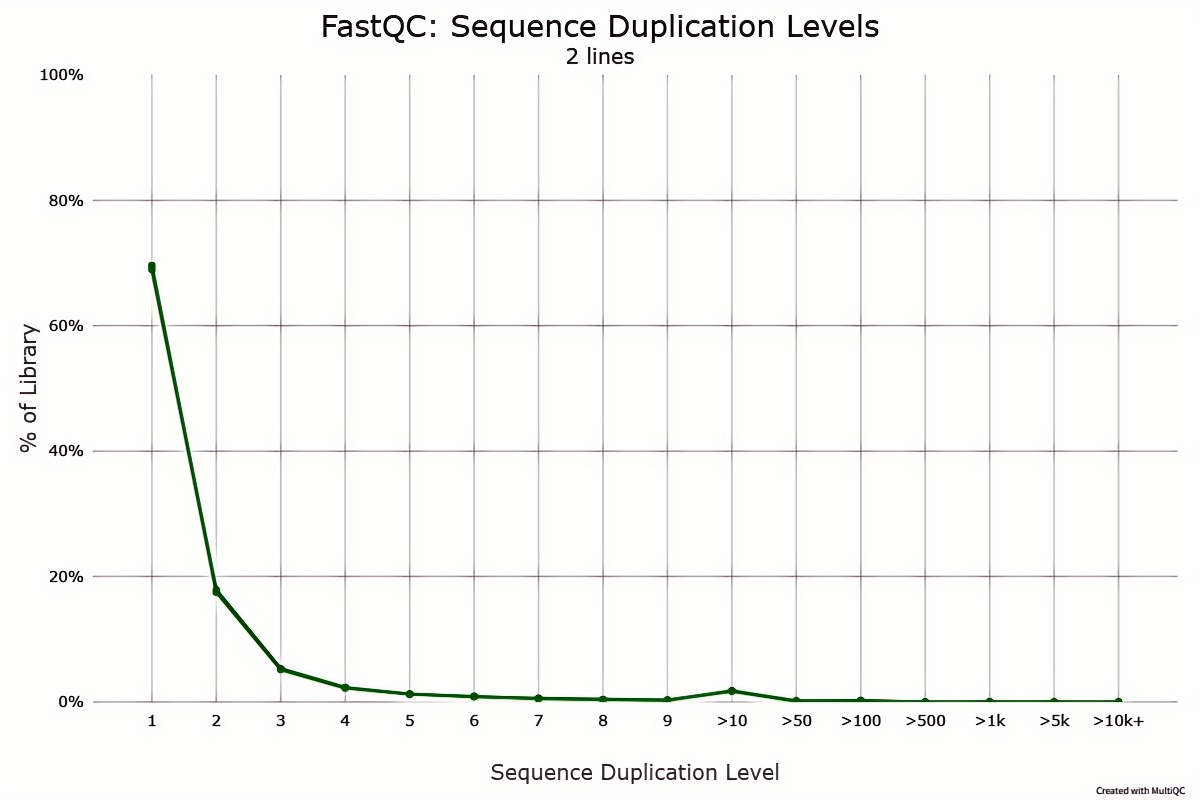


**Supplementary Figure 3.** Analysis of sequence duplication levels. Most sequences (approximately 69%) were unique, and the distribution of duplicates conformed to the expected exponential decay pattern, indicating the absence of significant technical bias from PCR amplification.


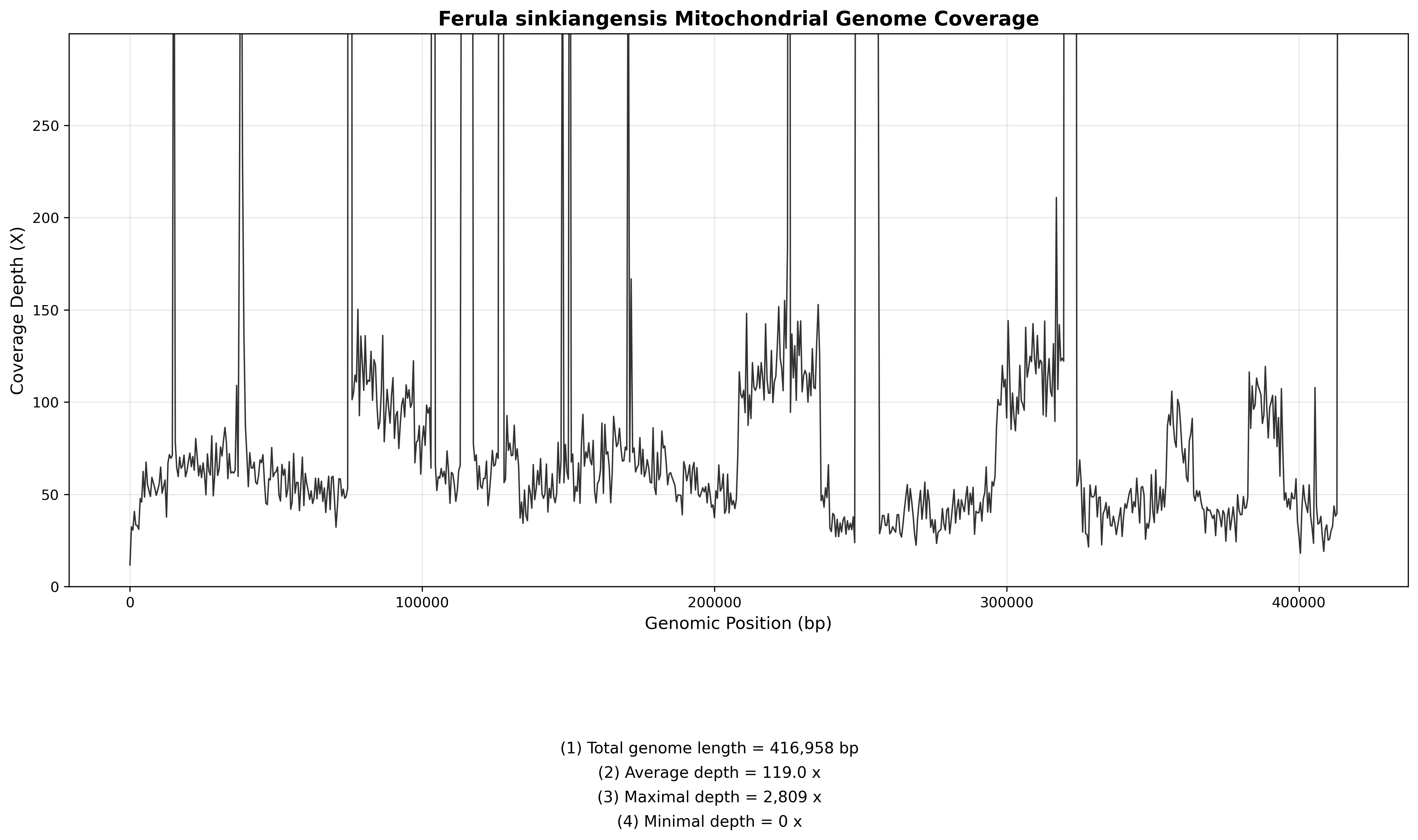


**Supplementary Figure** 4. Sequencing coverage depth across the complete mitochondrial genome assembly of F. sinkiangensis. The plot shows the depth of coverage (y-axis) for each nucleotide position (x-axis) along the 416,958 bp mitochondrial genome. The average sequencing depth across the entire genome was 119.0X. Regions with zero coverage (minimum depth = 0X) and regions with exceptionally high coverage (maximum depth = 2,809X) are common features of plant mitochondrial genomes, often corresponding to unique, non-coding regions and repetitive or multi-copy sequences, respectively.


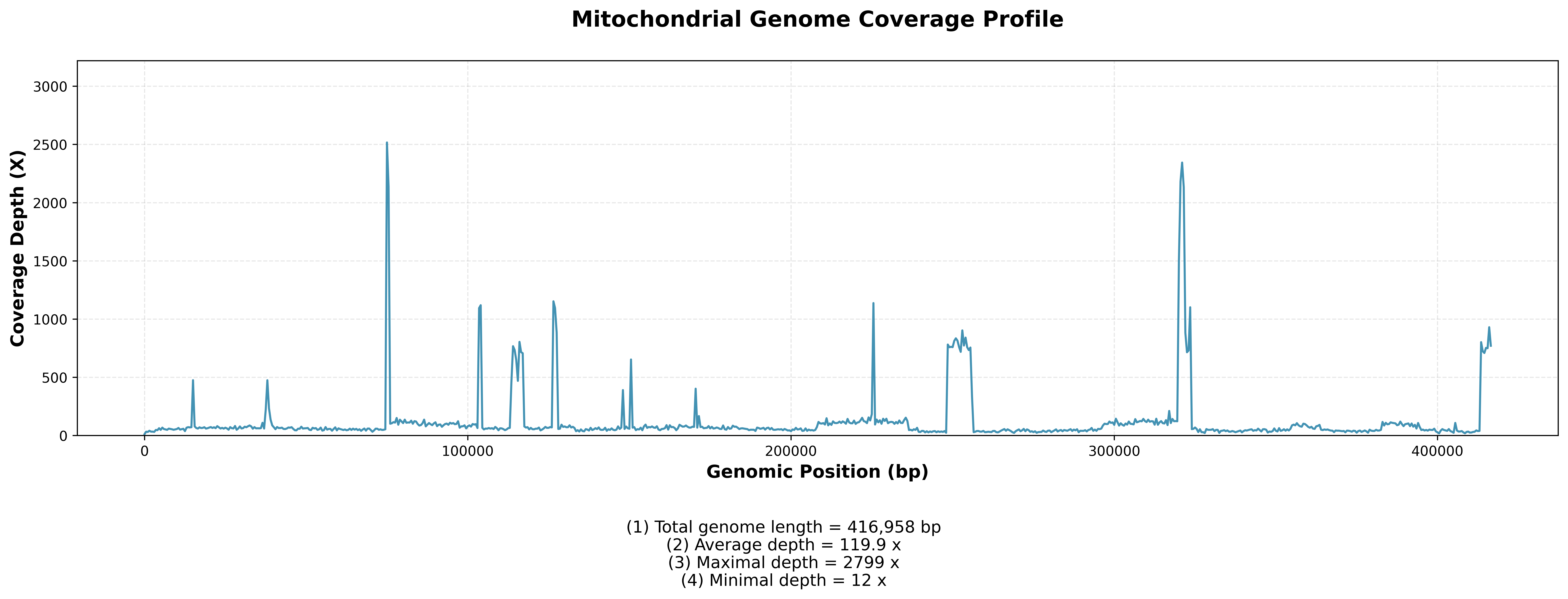


**Supplementary Figure 5.** Depth of coverage analysis of the mitochondrial genome assembly, emphasizing regions containing repetitive sequences. The plot illustrates the sequencing depth (y-axis) at each position (x-axis) in the 416,958 bp mitochondrial genome. The wide range of coverage depths, from a minimum of 12X to a maximum of 2,799X, with an average of 119.9X, reflects the structurally complex and repetitive structure of the plant mitochondrial DNA. Peaks of exceptionally high depth are consistent with the presence of repetitive or multi-copy sequences.


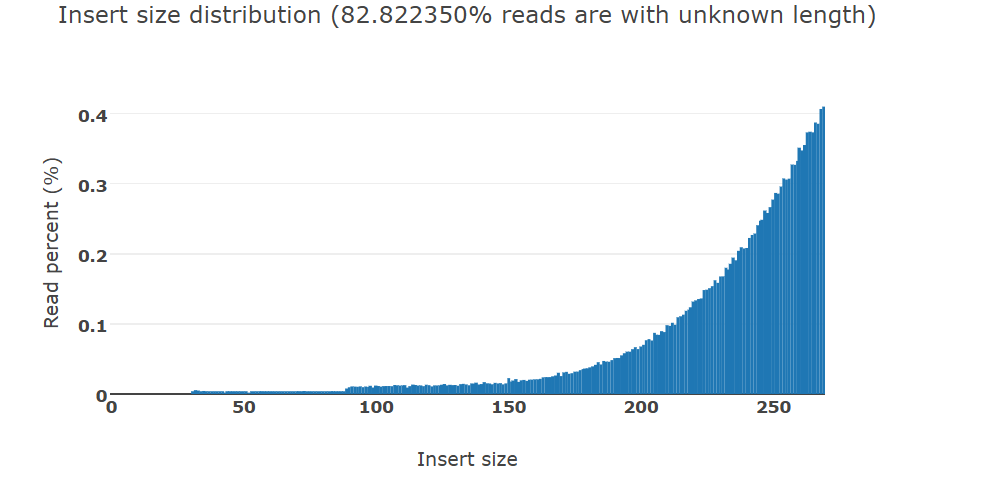


**Supplementary Figure 6.** Distribution of inferred insert sizes for the paired-end sequencing library. The plot shows the percentage of read pairs (y-axis) mapped to each inferred insert size (x-axis). The prominent single peak represents the most frequent insert fragment length. A large proportion of read pairs (82.82%) could not be assigned a precise insert size ("categorized as 'unknown'"), a common occurrence for sequences derived from repetitive regions, multi-copy organelles (e.g., chloroplasts and mitochondria), or other complex genomic regions that challenge unambiguous alignment.

**
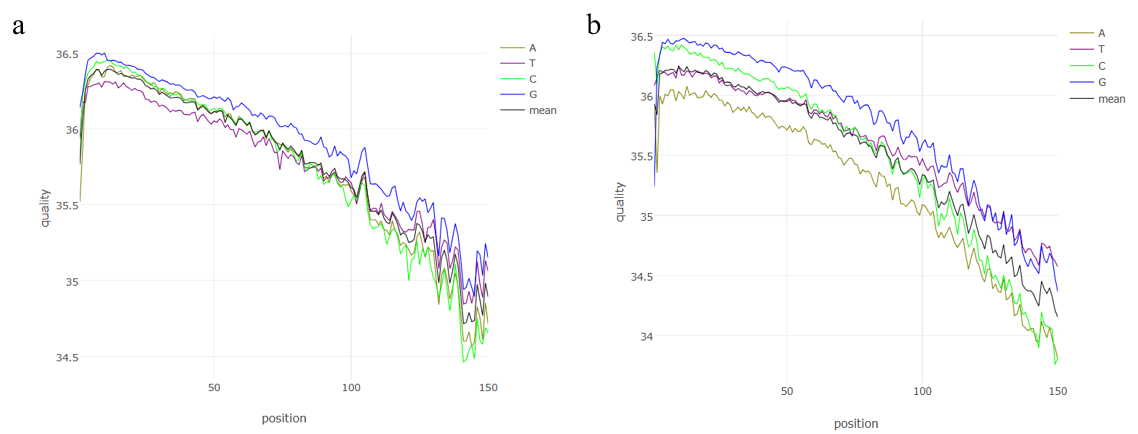
 Supplementary Figure 7.** Per-base sequencing quality across read positions for (a) Read 1 and (b) Read 2. The plots illustrate the average Phred quality scores for individual nucleotides (A, G, C, and T) and their overall mean (bold line) at each position along the sequencing read. Both forward (Read 1) and reverse (Read 2) reads demonstrate consistently high sequencing quality, with all nucleotide-specific quality profiles and the mean quality levels well above the Q30 threshold throughout the entire read length. The close overlap of the A, G, C, and T curves indicates a uniform base-calling accuracy across nucleotides, without a substantial base-specific bias.

**
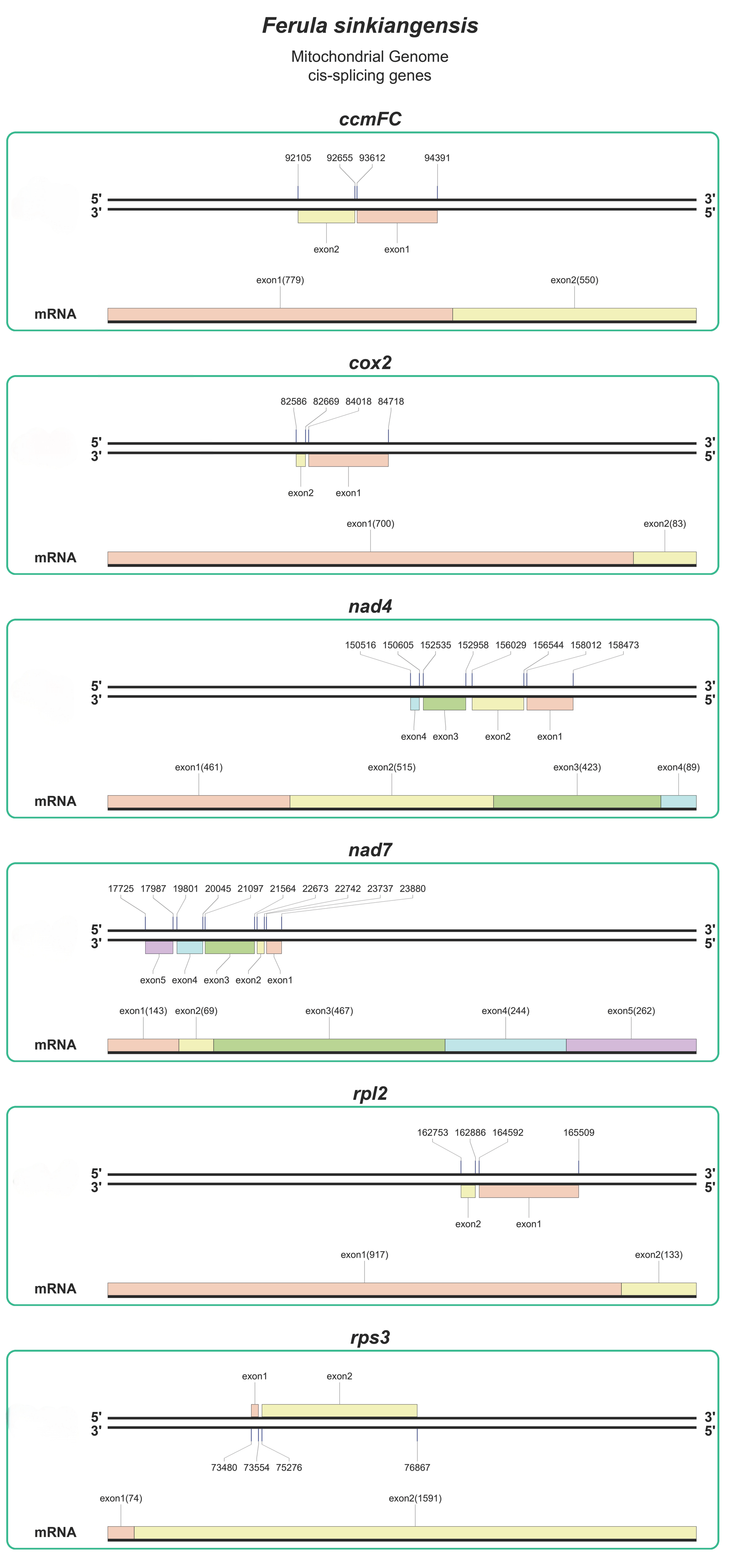
**

**Supplementary Figure 8.** Schematic representation of the cis-splicing genes in the mitochondrial genome. Exons and introns are denoted by different colors. The 5’ and 3’ labels indicate the sense direction of the gene. Note that the exon and intron lengths are not drawn to scale.

**
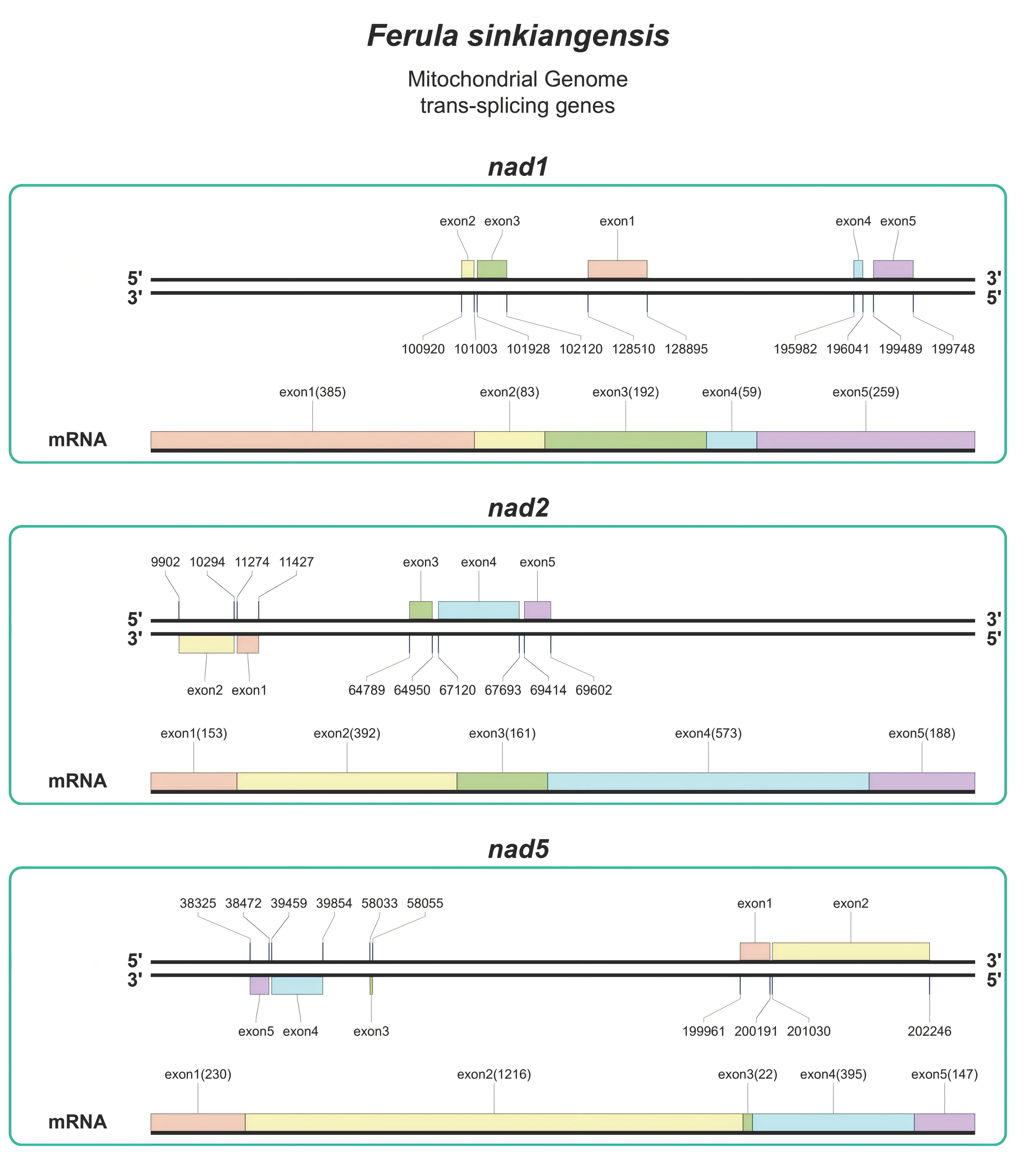
**

**Supplementary Figure 9.** Schematic representation of trans-splicing genes in the mitochondrial genome. Exons and introns are denoted by different colors. The labels 5' and 3' indicate the sense direction of the gene. Note that the exon and intron lengths are not drawn to scale.


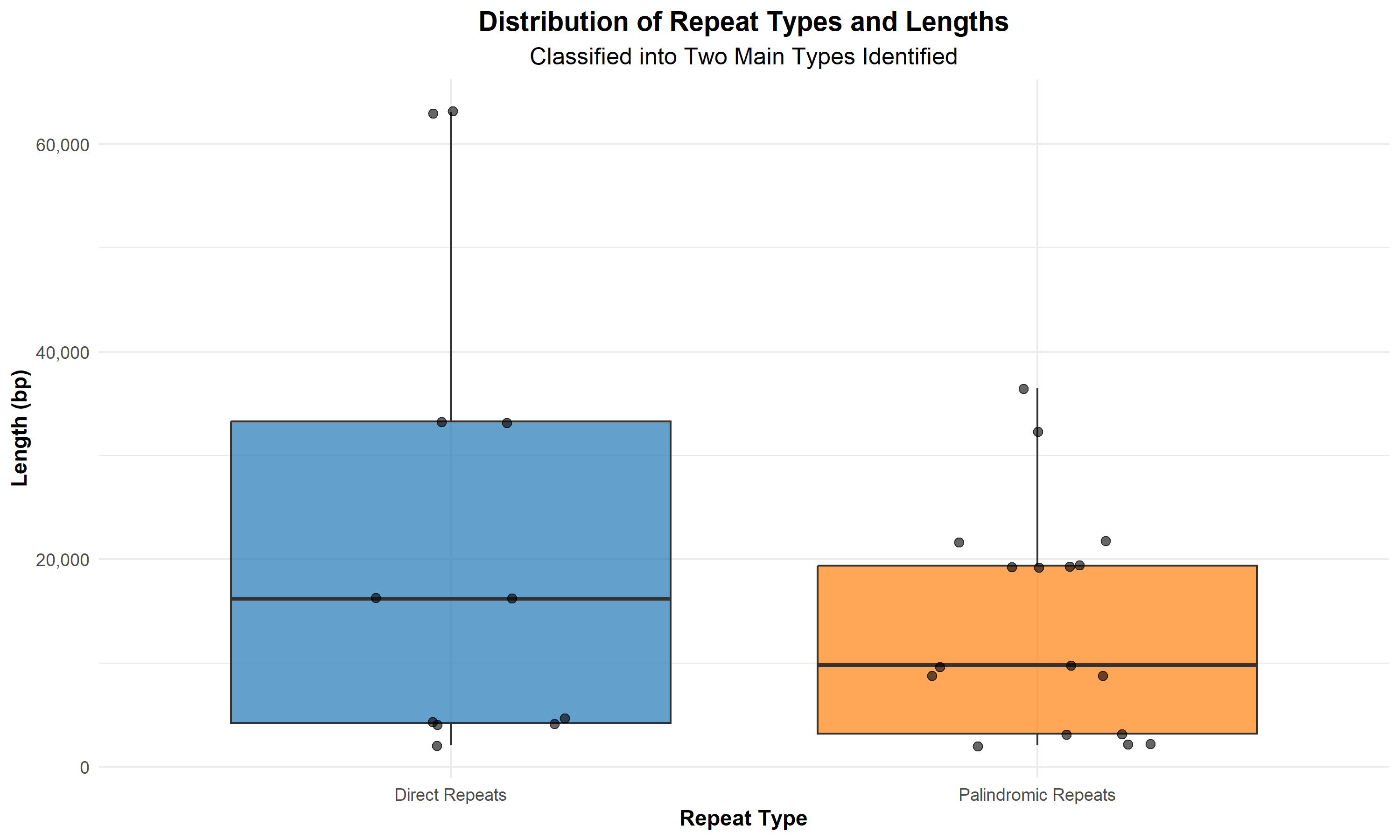


**Supplementary Figure 10.** Complete map of the 28 large repeat sequences (≥ 1 kb) identified within the mitochondrial genome.


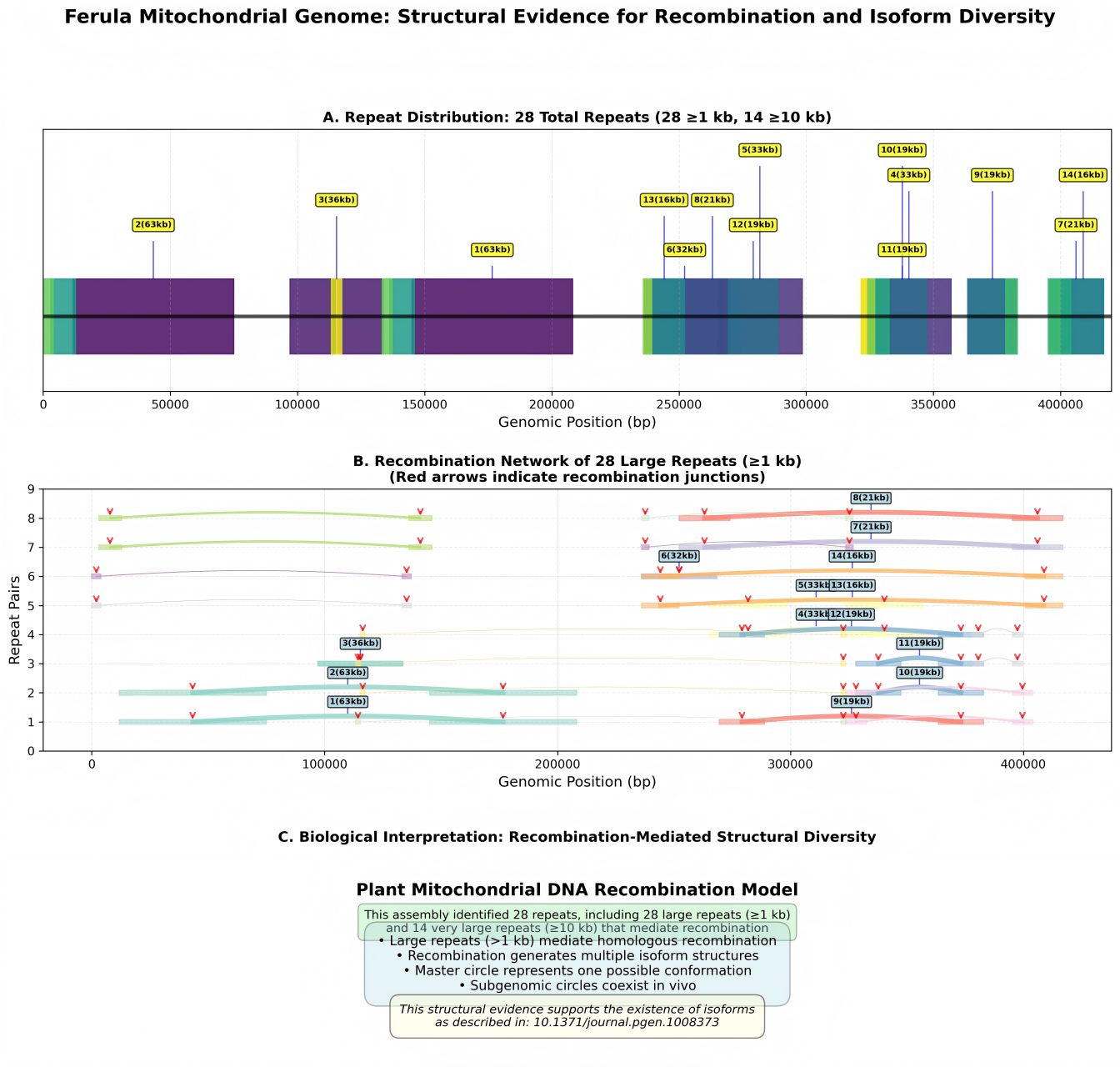


**Supplementary Figure 11.** Structural evidence for recombination-mediated isoform diversity in the *Ferula sinkiangensis* mitochondrial genome. (A) Linear map of the genome showing the distribution of repetitive sequences. The map illustrates the locations of all 28 identified repeat pairs (≥1 kb) within the assembled genome. Fourteen large repeat pairs (≥10 kb) are highlighted with connecting lines, representing the putative hotspots for homologous recombination. (B) Potential recombination network mediated by repetitive sequences. This model, constructed from the data in (A), illustrates the potential connections between genomic isoforms that could be facilitated by homologous recombination, with each arc representing a repeat pair. The red arrows indicate the putative recombination junctions validated by sequencing reads. The presence of these junctions was corroborated by uniform read coverage (average coverage 119.9×; Supplementary Figure 5).


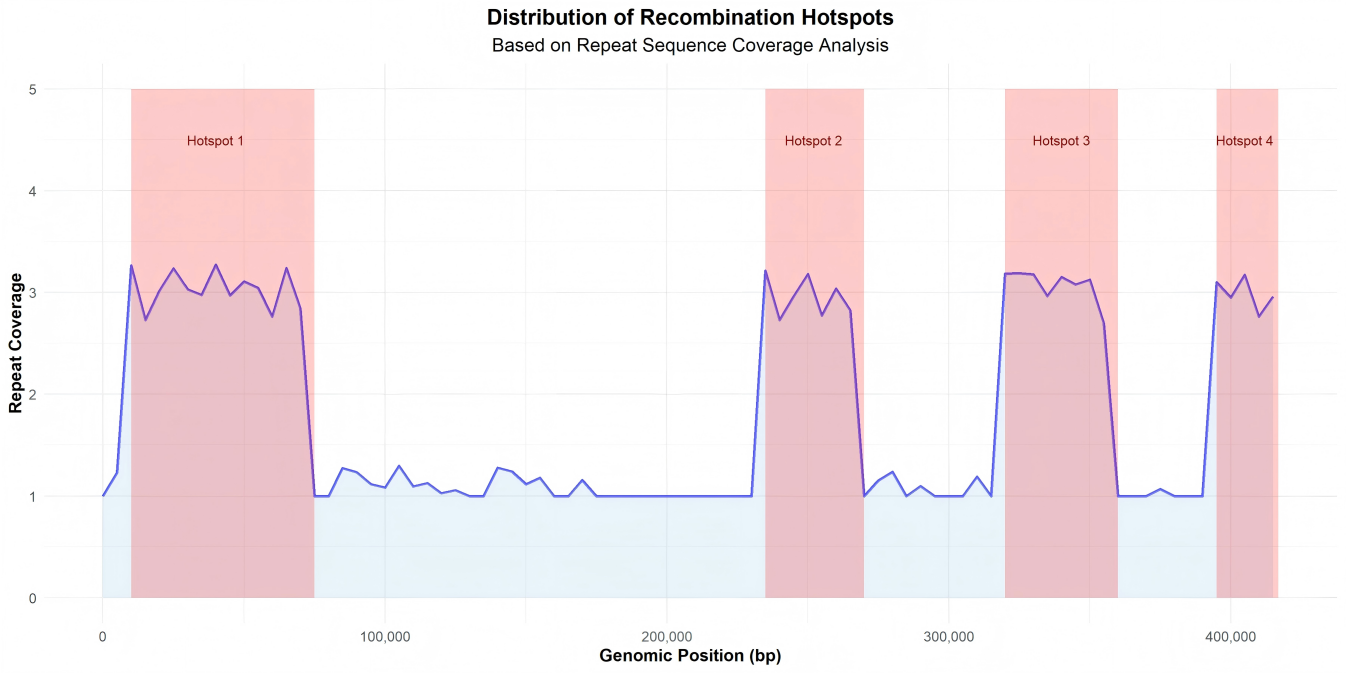


**Supplementary Figure 12.** Genome-wide recombination hotspot map. Potential recombination hotspots, inferred from the clustering of large repeat boundaries, are highlighted in red.


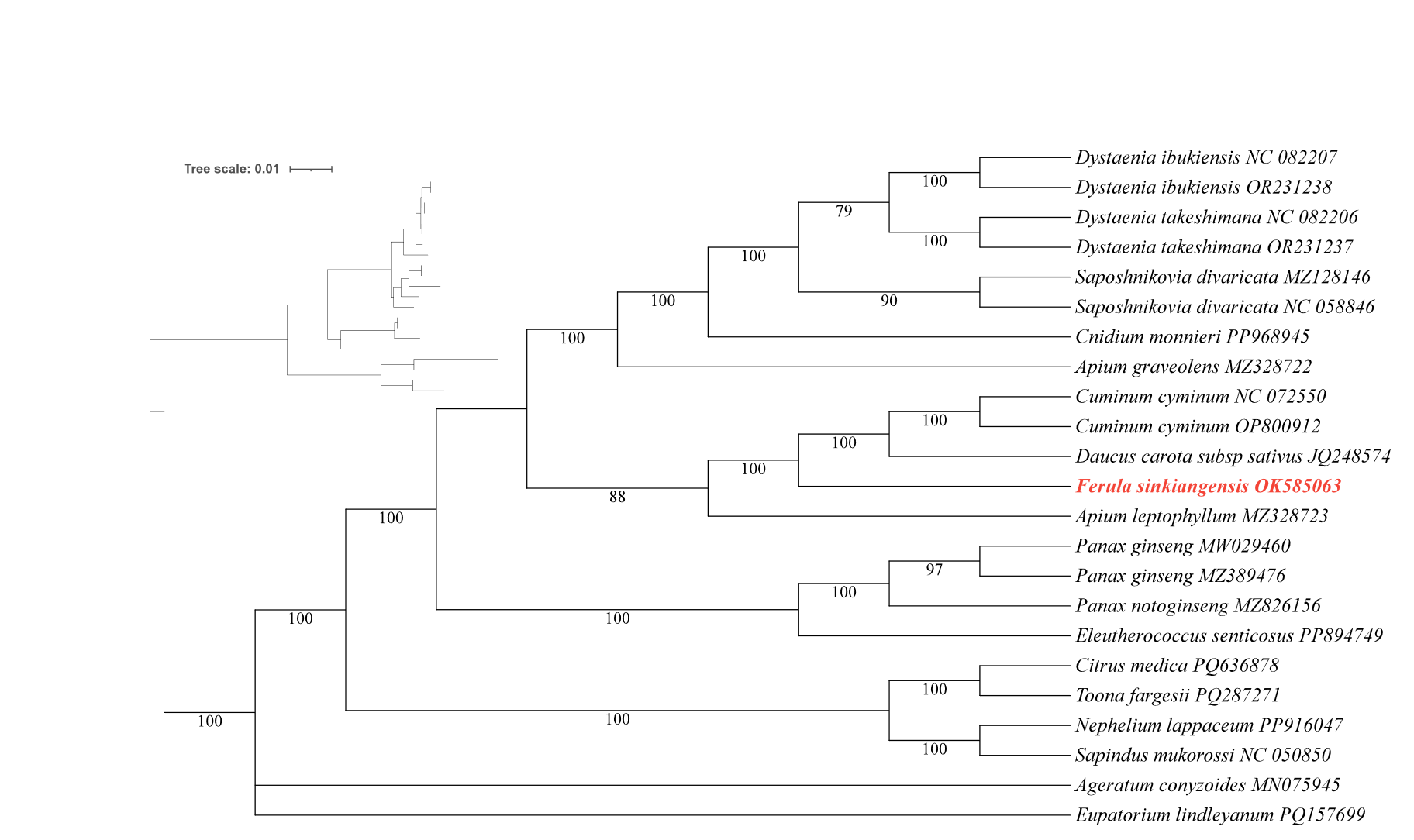


**Supplementary Figure 13.** Phylogenetic relationships of *F. sinkiangensis* and related taxa inferred from complete mitochondrial genome sequences using a Partition Model. The figure comprises two panels: a scale bar indicating branch lengths and a detailed phylogenetic tree. The support values indicated on the branches represent the bootstrap values from the partition model analysis. The target species are highlighted in bold red.


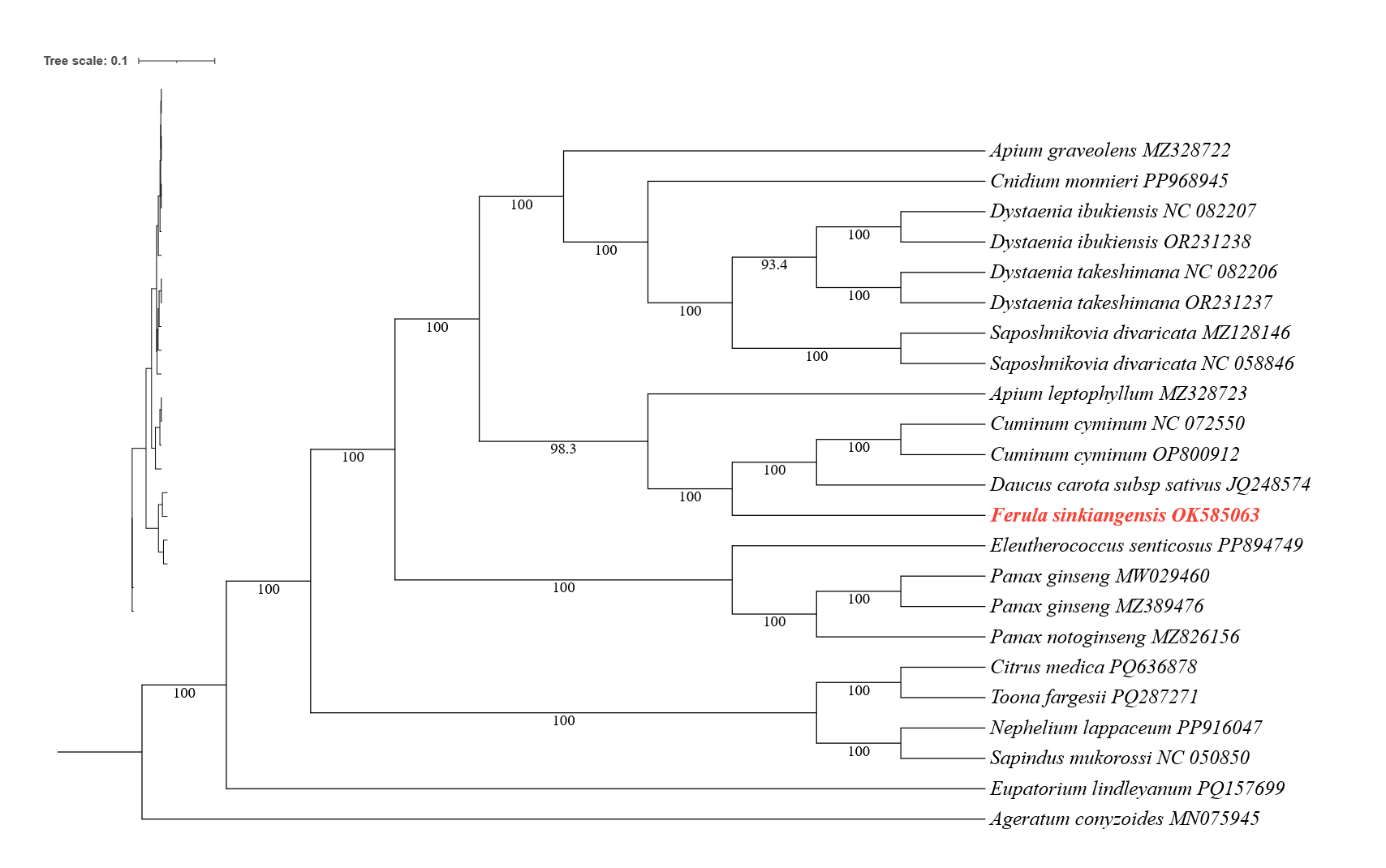


**Supplementary Figure 14.** Phylogenetic relationships of *F. sinkiangensis* and related taxa inferred from complete mitochondrial genome sequences using BI. The figure comprises two panels: a scale bar indicating branch lengths and the detailed phylogenetic tree. The support values indicated on the branches represent the posterior probabilities from the BI analysis. The target species are highlighted in red and bold.

| **Species** | **Voucher** | **Accession number** | **Authors and year** |
| --- | --- | --- | --- |
| *Ferula sinkiangensis* | 654021120525001LY | **OK585063** | **Fan et al. 2023** |
| *Dystaenia ibukiensis* | Genbank | NC_082207 | Park et al. 2023^#^ |
| *Dystaenia ibukiensis* | Genbank | OR231238 | Park et al. 2023^#^ |
| *Dystaenia takesimana* | Genbank | NC_082206 | Park et al. 2023^#^ |
| *Dystaenia takesimana* | Genbank | OR231237 | Park et al.2023^#^ |
| *Cnidium monnieri* | Genbank | PP968945 | Luo et al. 2024^#^ |
| *Cuminum cyminum* | Genbank | NC_072550 | Huang et al. 2023^#^ |
| *Cuminum cyminum* | Genbank | OP800912 | Huang et al. 2023^#^ |
| *Saposhnikovia divaricata* | Genbank | NC_058846 | Ni et al. 2023^#^ |
| *Saposhnikovia divaricata* | Genbank | MZ128146 | Ni et al. 2021^#^ |
| *Daucus carota* subsp. *sativus* | Genbank | JQ248574 | Iorizzo et al. 2012 |
| *Apium graveolens* | Genbank | MZ328722 | Li et al. 2021^#^ |
| *Apium leptophyllum* | Genbank | MZ328723 | Li et al. 2020^#^ |
| *Panax ginseng* | Genbank | MW029460 | Woojong Jang et al. 2020 |
| *Panax ginseng* | Genbank | MZ389476 | Jo et al. 2021^#^ |
| *Panax notoginseng* | Genbank | MZ826156 | Jo et al. 2021^#^ |
| *Eleutherococcus senticosus* | Genbank | PP894749 | Luo et al. 2024^#^ |
| *Citrus medica* | Genbank | PQ636878 | Li et al. 202^4#^ |
| *Sapindus mukorossi* | Genbank | NC_050850 | Chen et al. 2023^#^ |
| *Toona fargesii* | Genbank | PQ287271 | Lv et al. 2024^#^ |
| *Nephelium lappaceum* | Genbank | PP916047 | Luo et al. 2024^#^ |
| *Eupatorium lindleyanum* | Genbank | PQ157699 | Xia et al. 2024^#^ |
| *Ageratum conyzoides* | Genbank | MN075945 | Luo and Pan 2019 |

**Supplementary Table 1.** List of mitochondrial genome sequence sources used in this study. The target species of this study are indicated in bold. All sequences are publicly available in the GenBank database. Note: The mitochondrial genome of the target species has been previously published by our group (2021).

| **Number** | **PCGS** | **Species** |
| --- | --- | --- |
| 1 | atp1 | 23 |
| 2 | atp4 | 23 |
| 3 | atp6 | 23 |
| 4 | atp8 | 23 |
| 5 | ccmC | 23 |
| 6 | cox2 | 23 |
| 7 | cox3 | 23 |
| 8 | matR | 23 |
| 9 | nad3 | 23 |
| 10 | nad4L | 23 |
| 11 | nad7 | 23 |
| 12 | nad9 | 23 |
| 13 | rpl10 | 23 |
| 14 | rps4 | 23 |

**Supplementary Table 2.** Fourteen protein-coding genes conserved across all analyzed mitochondrial genomes. These 14 PCGs were identified as common to all twenty-three mitochondrial genomes included in the phylogenetic analysis. The detailed extraction steps are described in lines 83–87 of the main text.

| **Analysis Step** | **Software (Version)** | **Function** | **Primary Command Line / Parameters** |
| --- | --- | --- | --- |
| Quality Control | FastQC (v0.11.8) | Raw read quality assessment | fastqc SRR14018637_1.fastq SRR14018637_2.fastq -o results/fastqc_reports fastp -i SRR14018637_1.fastq -I SRR14018637_2.fastq -o SRR14018637_1_clean.fq -O SRR14018637_2_clean.fq --qualified_quality_phred 20 --length_required 50 --thread 2 --detect_adapter_for_pe --html fastp_report.html |
|  | Fastp (0.19.5) | Quality control and adapter removal |  |
|  | R (3.6.1) | Quality statistics and visualization |  |
|  | Kraken2 (2.0.9) | Organelle identification |  |
|  | GetOrganelle (1.6.4) | Mitochondrial genome assembly |  |
| Mitogenome Assembly | metaSPAdes (3.13.0) | GetOrganelle assembly engine | get_organelle_from_reads.py -1 SRR14018637_1_trimmed_paired.fq -2 SRR14018637_2_trimmed_paired.fq -o getorganelle_results -t 8 -k 117 -s Ferula_seed_genes.fasta --max-reads 1e9 -F mitochondrion |
|  | BWA (v0.7.17) | Plastid sequence removal and coverage calculation |  |
| Removal of Plastid Contamination | Samtools (1.12) | Read mapping and coverage calculation | bwa index Ferula_chloroplast_reference.fasta bwa mem -t 8 Ferula_chloroplast_reference.fasta SRR14018637_1_trimmed_paired.fq SRR14018637_2_trimmed_paired.fq | samtools view -b -f 4 -@ 8 - | samtools fastq -1 non_plastid_R1.fq -2 non_plastid_R2.fq -@ 8 - |
| Coverage Analysis | blast+ (2.9.0) | Sequence alignment | bwa index Ferula_mitochondrion_assembly.fasta bwa mem -t 8 Ferula_mitochondrion_assembly.fasta non_plastid_R1.fq non_plastid_R2.fq | samtools sort -@ 8 -o mitochondrion_specific_sorted.bam - samtools depth -aa mitochondrion_specific_sorted.bam > mitochondrion_coverage.txt |
|  | R (4.3) | Statistical analysis and visualization / Statistical analysis and plotting |  |
| Repeat Sequence Analysis | R (4.3) | Statistical analysis and visualization / Statistical analysis and plotting | makeblastdb -in Ferula_sinkiangensis_mitochondrion.fasta -dbtype nucl -out mito_db blastn -query Ferula_sinkiangensis_mitochondrion.fasta -db mito_db \  -outfmt "6 qseqid sseqid pident length mismatch gapopen qstart qend sstart send evalue bitscore" \  -evalue 1e-5 -word_size 11 -out self_alignment.txt awk '$4 >= 1000 && $3 >= 90 && $1 != $2' self_alignment.txt > large_repeats.txt |
|  | blast+ (2.9.0) | Sequence alignment |  |
| Phylogenetic Analysis | PhyloSuite (v1.2.3) | For extracting shared protein-coding genes | iqtree -s concatenation.phy -m MFP -bb 1000 -T AUTO  beast -beagle_SSE beast_run_simple.xml  treeannotator -burnin 10 -heights median beast_run_simple.trees beast_MCC_simple.tree  my_tree <- read.tree("beast_MCC_simple.tree") |
|  | IQ-TREE (2.2.0) | Sequence concatenation and partition model selection |  |
|  | PartitionFinder2 (2.1.1) | Bayesian Inference |  |
|  | BEAST2 (2.6.3) |  |  |
|  | TreeAnnotator (2.6.3) | Generating Maximum Clade Credibility (MCC) tree |  |
|  | - R: ape (5.7) | For results analysis and visualization |  |
|  | - R: phytools (2.0) | Statistical analysis and visualization / Statistical analysis and plotting |  |

**Supplementary Table 3.** Software versions and key command-line parameters used for bioinformatic analysis. This table details the key computational tools, their versions, and essential commands used from the initial quality assessment of raw sequencing data to phylogenetic tree construction, thereby ensuring methodological reproducibility.

| **total reads** | **total base** | **Q20 base** | **Q30 bases** | **Q40 bases** | **GC content** |
| --- | --- | --- | --- | --- | --- |
| 25.034578 M | 3.746327 G | 3.643193 G (97.247070%) | 3.443608 G (91.919592%) | 0 (0.000000%) | 35.80% |

**Supplementary Table 4.** Summary of sequencing quality-control metrics. Quality control and adapter trimming were performed using Fastp v0.11.8 to remove reads containing >3 N bases, with Q20 scores <60%, or shorter than 60 bp in length. This table presents the summary statistics of the cleaned sequencing data. A total of 25.03 million reads (3.75 Gb) were obtained. The data exhibited high base-call accuracy, with 97.25% and 91.92% of the bases achieving quality scores of ≥Q20 and ≥Q30, respectively. No base was called at Q40. The overall GC content was 35.80%.

| **Number** | **Repeat ID** | **Type** | **Position1** | **Position2** | **Length/bp** | **Similarity** | **E-value** |
| --- | --- | --- | --- | --- | --- | --- | --- |
| 1 | Repeat_1 | Forward | 145,027-208,140 | 11,783-74,896 | 63114 | 100% | 0.00e+00 |
| 2 | Repeat_2 | Forward | 11,783-74,896 | 145,027-208,140 | 63114 | 100% | 0.00e+00 |
| 3 | Repeat_3 | Palindromic | 97,087-133,585 | 133,585-97,087 | 36500 | 100% | 0.00e+00 |
| 4 | Repeat_4 | Forward | 323,718-356,978 | 265,181-298,441 | 33261 | 100% | 0.00e+00 |
| 5 | Repeat_5 | Forward | 265,181-298,441 | 323,718-356,978 | 33261 | 100% | 0.00e+00 |
| 6 | Repeat_6 | Palindromic | 236,054-268,381 | 268,381-236,054 | 32328 | 100% | 0.00e+00 |
| 7 | Repeat_7 | Palindromic | 395,185-416,958 | 273,991-252,218 | 21774 | 100% | 0.00e+00 |
| 8 | Repeat_8 | Palindromic | 252,218-273,991 | 416,958-395,185 | 21774 | 100% | 0.00e+00 |
| 9 | Repeat_9 | Palindromic | 363,487-382,882 | 288,884-269,489 | 19396 | 100% | 0.00e+00 |
| 10 | Repeat_10 | Palindromic | 363,487-382,882 | 347,421-328,026 | 19396 | 100% | 0.00e+00 |
| 11 | Repeat_11 | Palindromic | 328,026-347,421 | 382,882-363,487 | 19396 | 100% | 0.00e+00 |
| 12 | Repeat_12 | Palindromic | 269,489-288,884 | 382,882-363,487 | 19396 | 100% | 0.00e+00 |
| 13 | Repeat_13 | Forward | 400,795-416,958 | 236,054-252,217 | 16164 | 100% | 0.00e+00 |
| 14 | Repeat_14 | Forward | 236,054-252,217 | 400,795-416,958 | 16164 | 100% | 0.00e+00 |
| 15 | Repeat_15 | Palindromic | 136,219-145,997 | 12,753-2,975 | 9779 | 100% | 0.00e+00 |
| 16 | Repeat_16 | Palindromic | 2,975-12,753 | 145,997-136,219 | 9779 | 100% | 0.00e+00 |
| 17 | Repeat_17 | Palindromic | 395,185-403,995 | 332,528-323,718 | 8811 | 100% | 0.00e+00 |
| 18 | Repeat_18 | Palindromic | 323,718-332,528 | 403,995-395,185 | 8811 | 100% | 0.00e+00 |
| 19 | Repeat_19 | Forward | 395,185-399,687 | 378,380-382,882 | 4503 | 100% | 0.00e+00 |
| 20 | Repeat_20 | Forward | 378,380-382,882 | 395,185-399,687 | 4503 | 100% | 0.00e+00 |
| 21 | Repeat_21 | Forward | 133,245-137,189 | 1-3,945 | 3945 | 100% | 0.00e+00 |
| 22 | Repeat_22 | Forward | 1-3,945 | 133,245-137,189 | 3945 | 100% | 0.00e+00 |
| 23 | Repeat_23 | Palindromic | 323,718-326,918 | 239,254-236,054 | 3201 | 100% | 0.00e+00 |
| 24 | Repeat_24 | Palindromic | 236,054-239,254 | 326,918-323,718 | 3201 | 100% | 0.00e+00 |
| 25 | Repeat_26 | Palindromic | 321,661-323,709 | 117,384-115,336 | 2049 | 100% | 0.00e+00 |
| 26 | Repeat_28 | Palindromic | 115,336-117,384 | 323,709-321,661 | 2049 | 100% | 0.00e+00 |
| 27 | Repeat_25 | Forward | 321,661-323,709 | 113,287-115,335 | 2049 | 100% | 0.00e+00 |
| 28 | Repeat_27 | Forward | 113,287-115,335 | 321,661-323,709 | 2049 | 100% | 0.00e+00 |

**Supplementary Table 5.** Identification of large repetitive sequences (≥1 kb) within the mitochondrial genome. The table lists all repetitive sequences with a length of ≥1 kb in descending order. The top 14 sequences are categorized as Large Repeats (≥10 kb). The repeat type "Forward" denotes direct repeats, whereas "Palindromic" denotes inverted (reverse complement) repeats.

| **Hotspot Region** | **Genomic Coordinates** | **Associated Repeats** | **Repeats Providing Junctions** | **Potential Recombination** |
| --- | --- | --- | --- | --- |
| Hotpot 1 | ~10,000 - 75,000 bp | Repeat_1,  Repeat_2,  Repeat_15,  Repeat_16,  Repeat_21,  Repeat_22 | Repeat_1/2 (63.1 kb) | Large-scale inversion/exchange |
| Hotpot 2 | ~235,000 - 270,000 bp | Repeat_6,  Repeat_13,  Repeat_14,  Repeat_23,  Repeat_24 | Repeat_6 (32.3 kb), Repeat_13/14 (16.2 kb) | Complex rearrangements mediated by inverted repeats |
| Hotpot 3 | ~320,000 - 360,000 bp | Repeat_4,  Repeat_5,  Repeat_17,  Repeat_18,  Repeat_23,  Repeat_24,  Repeat_25,  Repeat_26,  Repeat_27,  Repeat_28 | Repeat_4/5 (33.3 kb), Repeat_17/18 (8.8 kb) | Homologous recombination, enabling multipath connections |
| Hotpot 4 | ~395,000 - 417,000 bp | Repeat_7,  Repeat_8,  Repeat_13,  Repeat_14,  Repeat_17, Repeat_18, Repeat_19,  Repeat_20 | Repeat_7/8 (21.8 kb), Repeat_13/14 (16.2 kb) | Terminal-associated recombination |

**Supplementary Table 6.** Characterization of recombination hotspots and their associated large repeats.This table details the four primary recombination hotspots identified in the *F. sinkiangensis* mitochondrial genome (Supplementary Figure 12). For each hotspot, the table lists the genomic coordinates, all associated large repeats (≥1 kb), the specific major repeat pairs inferred to generate the predominant recombination junctions, and the potential structural rearrangement mechanisms mediated by these repeats.
